# Effects of altitude on circadian rhythm of adult locomotor activity in Himalayan strains of *Drosophila helvetica*

**DOI:** 10.1186/1740-3391-5-1

**Published:** 2007-01-09

**Authors:** Keny Vanlalhriatpuia, Vanlalnghaka Chhakchhuak, Satralkar K Moses, SB Iyyer, MS Kasture, AJ Shivagaje, Barnabas J Rajneesh, Dilip S Joshi

**Affiliations:** 1 Zoology Department, Ahmednagar College, Ahmednagar 414001, M.S., India

## Abstract

**Background:**

We recently reported that the altitude of origin altered the photic and thermal sensitivity of the circadian pacemaker controlling eclosion and oviposition rhythms of high altitude Himalayan strains of *Drosophila ananassae*. The present study was aimed at investigating the effects of altitude of origin on the pacemaker controlling the adult locomotor activity rhythm of *D. helvetica*.

**Methods:**

Locomotor activity rhythms of the high altitude Himalayan (*haH*) strain (Hemkund-Sahib, 4,121 m above sea level) and the low altitude Himalayan (*laH*) strain (Birahi, 1,132 m a.s.l.) of *D. helvetica *were assayed by two experiments. The first experiment examined the natural entrainment pattern in light-dark (LD) cycles at the breeding site of each strain. The second experiment examined the entrainment parameters in LD 12:12 cycles and the period of free-running rhythm in constant darkness (DD) under controlled laboratory conditions.

**Results:**

When entrained by natural or artificial LD cycles, the *haH *strain had an unimodal activity pattern with a single peak that commenced in the forenoon and continued till evening, while the *laH *strain had a bimodal activity pattern in which the morning peak occurred before lights-on and was separated by about 4 h from the evening peak. Unimodality of the *haH *strain was retained in DD; however, bimodality of the *laH *strain was abolished in DD since the evening peak disappeared immediately after the trasfer from LD 12:12 to DD. The period of the free-running rhythm of the *haH *strain was ~26.1 h, whereas that of the *laH *strain was ~21.7 h.

**Conclusion:**

Parameters of entrainment and free-running rhythm of the adult locomotor activity of the *haH *strain of *D. helvetica *were strikingly different from those of the *laH *strain and were likely due to ecological adaptations to the prevailing environmental conditions at the altitude where the species evolved.

## Background

Latitude and altitude of origin are known to modify fundamental properties of the pacemaker controlling circadian rhythms of eclosion, oviposition and adult locomotor activity of naturally occurring clock phenotypes of *Drosophila *[1]. For example, latitude of origin dramatically altered the basic parameters of eclosion rhythm of the Japanese strains of *D. auraria *[2,3], the European strains of *D. littoralis *[4-6] and *D. subobscura *[7]. Variability in properties of eclosion and adult locomotor activity rhythms of *D. ananassae *strains captured from Sri Lanka and India were accounted for by the environmental conditions prevailing at the latitude of origin [8,9]. Molecular polymorphism in the *period *gene of latitudinal strains of *D. melanogaster*, *D. littoralis *and *D. simulans *captured in Europe, Africa and Australia was ascribed to natural selection [1,10-13]. Variation in patterns of adult locomotor activity of eleven species of *Drosophila *originating from across the United States were attributed to the latitude of origin and the physical nature of the breeding site [14].

Studies on the altitudinal variation in the circadian physiology of *Drosophila *are rather few as compared to those on latitudinal variation. For example, altitudinal variation in photoperiodic response to diapause and the number of generations per year were studied in strains of four *Drosophila *species from Japan [15]. Genetic components for the altitudinal differences in oviposition rhythm were analyzed by carrying out crosses within and between populations of *D. buzzatii *that originated from different altitudes in Argentina [16]. Altitude of origin also modified the pacemaker properties controlling eclosion and oviposition rhythms of *D. ananassae *strains [17-21]. The present experiments were designed to determine whether or not the altitude of origin affected the parameters of entrainment and free-running rhythm of adult locomotor activity of *D. helvetica *strains originating from different altitudes in the Himalayas.

## Methods

Laboratory populations of the high altitude Himalayan (*haH*) strain of *D. helvetica *(Burla, 1948) originating from Hemkund-Sahib (4,121 m above sea level, 30.81°N, 79.81°E) and the low altitude Himalayan (*laH*) strain originating from Birahi (1,132 m a.s.l., 30.62°N, 79.65°E) were derived from 21 and 15 wild-caught gravid females in the field, respectively, in April 2005. Thus, they were genetic representatives of the populations from their breeding sites in nature. Flies were maintained on standard cornmeal medium at 20°C (± 0.5°C) and ~60% relative humidity under cycles of 12 h of white light at 100 lux and 12 h of complete darkness (LD 12:12). Males of these strains were used for assaying the adult locomotor activity in the field and laboratory. The activity rhythm of individual flies was monitored by the computerized method described elsewhere in detail [8]. The method in brief is as follows: Activity of an individual male was monitored by placing the fly into a glass tube (length × outer diameter: 100 × 7 mm). One end of the tube was inserted into a container having 10 g of culture medium and the other end was plugged with cotton. The tube was placed in the path of an infrared beam. The culture medium was replenished once a week. Interruption of the infrared beam by fly movement triggered an all-or-none electronic signal that was amplified, counted, accumulated and then registered every 6 min on the hard disk of a computer.

Onset of activity was considered as the phase reference point of the rhythm because it was a more precise and predictable phase point than the mid-point or end of activity. The phase of activity onset (Ψ_o_) was defined as the time from the sunrise in the field or lights-on of LD 12:12 cycles in the laboratory to the time of activity onset as given by an eye-fitted line to 11 successive activity onsets. The phase of activity termination (Ψ_e_) was defined as the time from the sunset in the field or lights-off of LD 12:12 cycles in the laboratory to the time of end of activity as given by an eye-fitted line to 11 successive activity offsets. The interval (Ψ_*M-E*_) between the morning peak and evening peak during entrainment was measured by eye-fitted lines to the offsets of morning peaks and onsets of evening peaks for 11 days. Activity phase (α) was regarded as the average interval between two eye-fitted lines joining 11 activity onsets and 11 activity offsets. The rest phase (ρ) was calculated by subtracting α from 24 h during entrainment or from the period of free-running rhythm (τ) in constant darkness (DD). τ was determined by fitting least square regression line to 11 successive activity onsets during steady-state free-runs. Total activity per cycle (*TAPC*) in each strain was determined by taking the average number of activity passes from the pooled data of 21 flies for 11 days during entrainment or free-running state. When entrained by natural LD 14.1:9.9 cycles in the field or artificial LD 12:12 cycles in the laboratory, the activity peaks of each strain were restricted to specific times of the photophase. Thus, it was possible to divide the natural photophase of 14.1 h or the artificial photophase of 12 h equally into three sectors: morning, midday and evening. A fly was considered to have the morning, midday and/or evening peak of activity if the activity passes in the given part of the photophase were > 30% of the *TAPC*.

Parameters of entrainment and free-running rhythm were assayed in 21 males (age: 3 days post-eclosion) of each strain in two sets of experiments. The first set of experiments was performed simultaneously at the breeding site of each strain in the field to study the natural entrainment patterns of these strains in LD cycles under two temperature regimes, the naturally fluctuating temperature (*NFT*) and at constant temperature (*CT*) of 20 ± 0.5°C. Initially five culture bottles (10 adults of each gender per bottle) and 21 activity monitoring units were housed in each of two identical glass-enclosures (2 × 2 × 2 m) which were uniformly exposed to direct sunlight at the breeding site of each strain. Cross ventilation in each glass-enclosure was achieved by two screened openings (15 × 15 cm). Maintenance (i.e., transferring flies to the bottles containing fresh culture medium, changing the culture medium in the containers of activity recording units, etc.) was carried out by manipulation through two sleeves attached to round openings (21 cm diameter) in each glass enclosure. Flies were bred for one generation and then entrainment was studied in adults of the next generation at the same temperature regime in which they were reared. Flies in the first glass enclosure were exposed to the *NFT *regime. Flies in the second glass enclosure were exposed to the *CT *at 20 ± 0.5°C by connecting both of the enclosure openings to a split air-conditioning machine whose thermostat was kept at 20°C. Relative humidity in all four glass enclosures was 60 ± 10%, as both breeding sites were surrounded by moist vegetation. Data on adult locomotor activity of both strains were obtained in July 2005 when the natural photoperiod was ~14 h. Temperature, relative humidity and light intensity were recorded continuously.

A second set of experiments examined the entrainment and free-running rhythm parameters at 20 ± 0.5°C and ~60 % relative humidity in the laboratory. Entrainment was studied in LD 12:12 cycles (100 lux during L and complete darkness during D) for 11 days, and then the flies were transferred to DD to determine τ. Effects of altitude and temperature on Ψ_o_, Ψ_e_, Ψ_*M-E*_, α/ρ ratio, *TAPC *and τ of both strains were compared by *t*-tests.

## Results

**Figure 1:**
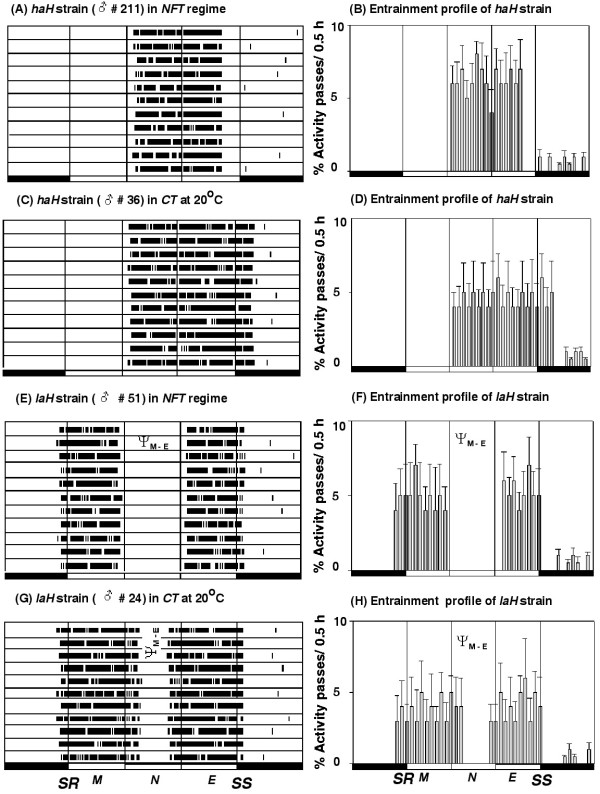
**Activity records of representative males of the *haH *and *laH *strains of *D. helvetica *monitored at the breeding site of each strain in the field in natural light-dark 14.1:9.9 h cycles under *NFT *and *CT *regimes**. Black portions of each time bar indicate the scotophase and the open portion indicates the photophase. *SR *and *SS *denote the sunrise and sunset, respectively, while *M*, *N *and *E *denote the morning, noon and evening, respectively. Onset of activity of both males of the *haH *strain occurred in the forenoon ~4.5 h after *SR *in both temperature regimes, but the end of activity of male # 211 (A) occurred ~1 h before SS in *NFT *regime, and that of the male # 36 (C) occurred ~1 h after *SS *in *CT *regime. Onset and end of activity in both males of the *laH *strain occurred ~1 h before *SR *and ~0.4 h after *SS *under both temperature regimes, but the Ψ_*M-E *_of the male # 51 (E) in *NFT *regime was 4.8 h, while that of the male # 24 (G) was 2.2 h in *CT *regime. The mean entrainment activity profile of 21 males of each strain for 11 days is shown in histogram format (B, D, F, H). Males of the *haH *strain had a unimodal activity pattern in both temperature regimes; however, activity ended ~1 h before *SS *in the *NFT *regime (B) and ~1 h after *SS *in the *CT *regime (D). Males of the *laH *strain had bimodal activity patterns in both temperature regimes; however, Ψ_*M-E *_was 5.2 h in *NFT *regime (F) and 2.3 h in the *CT *regime (H).

**Figure 2:**
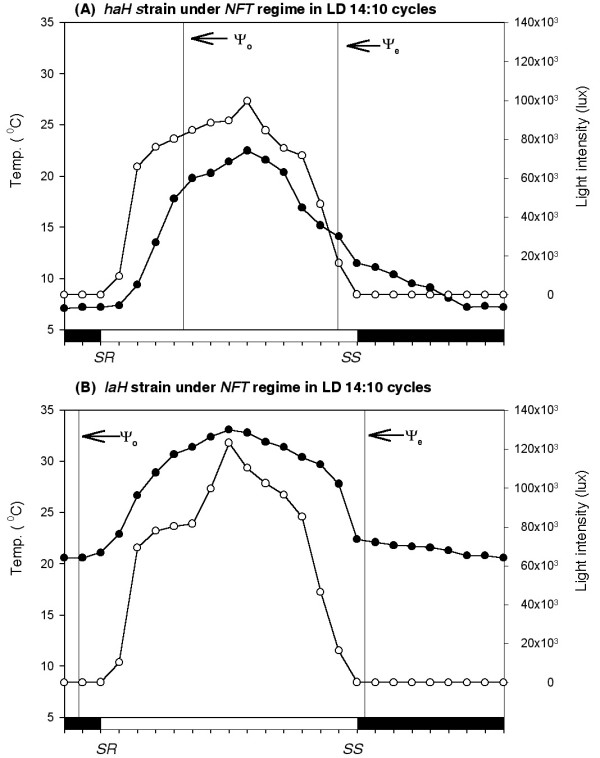
**Curves passing through mean values (*N *= 22) of the natural light intensity (open circles) and environmental temperature (filled circles) obtained at the breeding site of the *haH *strain (A) at Hemkund-Sahib (4,121 m) and at the breeding site of the *laH *strain of *D. helvetica *(B) at Birahi (1,132 m)**. Data were collected from 1 to 21 July 2005 when the natural photoperiod was ~14 h. Note that light intensity curves were almost similar at both breeding sites but the temperature curves were strikingly dissimilar. Ψ_o_and Ψ_e _(vertical lines) denote the phase of activity onset and the phase of activity offset, respectively. The *haH *flies began activity ~4.5 h after sunrise (*SR*) when the temperature and light intensity were ~19°C and 79,000 lux, respectively, while the *laH *flies began activity ~1 h before sunrise when the temperature and light intensity were ~20°C and 1 lux, respectively. The *haH *flies terminated activity 1.4 h before sunset (*SS*) when temperature and light intensity were ~14°C and 17,000 lux, respectively, while the *laH *flies terminated activity ~0.4 h after sunset when the temperature and light intensity were ~22°C and 30 lux, respectively.

**Figure 3:**
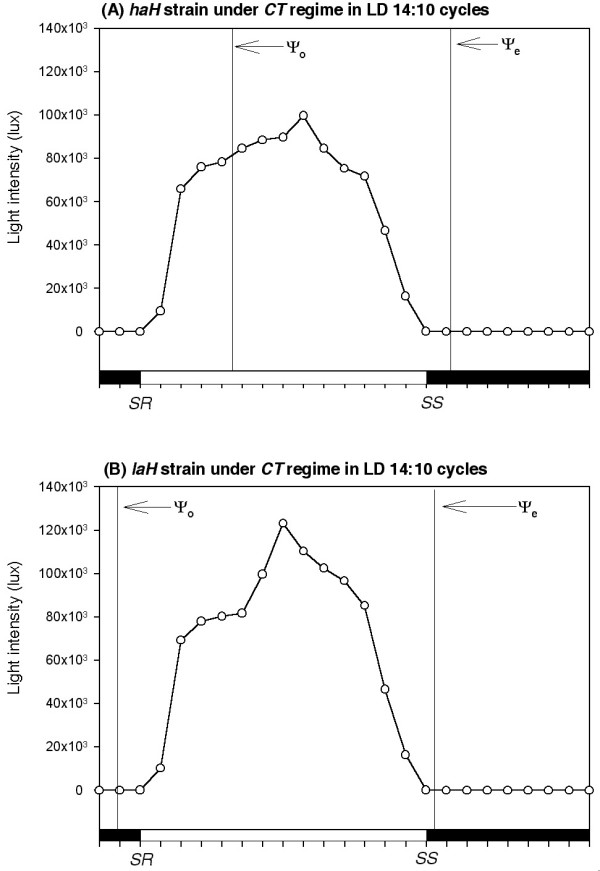
**Curves passing through mean values (*N *= 22) of the natural light intensity obtained at the breeding site of the *haH *strain (A) at Hemkund-Sahib (4,121 m) and the *laH *strain of *D. helvetica *(B) at Birahi (1,132 m)**. Data were collected from 1 to 21 July 2005 when the natural photoperiod was ~14 h and the ambient temperature was maintained at 20 ± 0.5°C, (i.e., the *CT *regime). The *haH *flies began activity ~4.5 h after sunrise (*SR*) when the light intensity was ~79,000 lux while the *laH *flies began activity ~1 h before sunrise when the light intensity was ~1 lux as in the *NFT *regime. The *haH *flies terminated activity ~1 h after sunset (*SS*) when light intensity was ~1 lux unlike that in the *NFT *regime, while the *laH *flies terminated activity ~0.4 h after sunset when the light intensity was ~30 lux as that in the *NFT *regime. Other symbols as in Figure 1.

Figure 1 illustrates the activity records of representative males of each strain in actogram format and the mean entrainment pattern of 21 males of each strain for 11 days in histogram format. Mean values of five entrainment parameters of these strains are given in Table 1. Although both strains were entrained by natural LD cycles in the *NFT *and *CT *regimes, the altitude of origin altered the uni/bimodality of the activity pattern (Figure 1) and the other four parameters of entrainment of the *haH *strain (Table 1). The *haH *strain had unimodal activity pattern with a single delayed peak that commenced in the forenoon and continued till evening, while the *laH *strain had bimodal activity pattern with early morning peak that was separated from the evening peak by a period of inactivity of ~4 h (Figure 1A–H). Figure 2 gives profiles for the natural light intensity and environmental temperature obtained at the breeding site of each strain in the *NFT *regime. Although light intensity profiles at the two breeding sites were almost similar, the temperature profiles were strikingly different, as the temperature varied from ~7 to 21°C at the high altitude (Figure 2A) but from ~20 to 33°C at the low altitude (Figure 2B). Figure 3 gives almost similar profiles for the natural light intensity obtained at the breeding sites of these strains under the *CT *regime at 20 ± 0.5°C. Ambient temperature did not affect the Ψ_o _of either strain and Ψ_e _of the *laH *strain but it affected the Ψ_e _of the *haH *strain as it was postponed by ~2 h in the *CT *regime (Figures 2 and 3, Table 1). Ambient temperature also affected the Ψ_*M-E *_of the *laH *strain as it was reduced by ~3 h in the *CT *regime as compare to that in the *NFT *regime (Figure 1E–H, Table 1). The reduction in Ψ_*M-E *_was caused by the addition of activity at the end of the morning peak and at the beginning of the evening peak (Figure 1G, H).

**Table 1:** Entrainment parameters of *D. helvetica *strains in the field

Strain	Ψ_o _(h)	Ψ_e _(h)	Ψ_*M-E *_(h)	α/ρ	*TAPC*
*haH *at *NFT*	5.2 (0.4)	- 1.4 (0.5)		0.4 (0.1)	310 (8)
*haH *at *CT*	5.2 (0.3)	1.1 (0.4)		0.7 (0.2)	601 (76)
*laH *at *NFT*	- 1.2 (0.3)	0.4 (0.3)	5.6 (0.4)	1.7 (0.4)	501 (49)
*laH *at *CT*	- 1.1 (0.3)	0.4 (0.2)	2.4 (0.4)	1.7 (0.4)	813 (39)

Mean values (± *SD*, *N *= 21) for the phase of activity onset (Ψ_o_), phase of activity end (Ψ_e_), the interval between morning and evening peaks (Ψ_*M-E*_), the ratio of activity phase to rest phase (α/ρ) and the total activity per cycle (*TAPC*) for males of the *haH *strain and the *laH *strain of *D. helvetica *entrained by natural LD 14.1:9.9 cycles in the naturally fluctuating temperature (*NFT*) regime and in constant temperature (*CT*) regime (20 ± 0.5°C) at the altitude of origin in the field.

**Figure 4:**
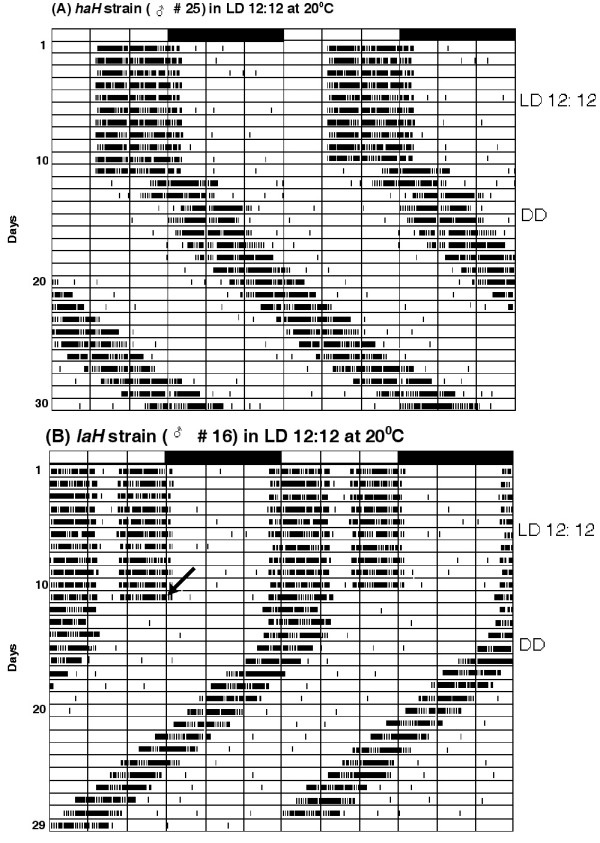
**Double plotted activity records of the representative male (# 25) of the *haH *strain (A) and the male (# 16) of the *laH *strain (B) of *D. helvetica *entrained by LD 12:12 cycles at 20 ± 0.5°C for 11 days under controlled laboratory conditions**. The male of the *haH *strain had unimodal activity pattern with onset of activity occurring in the subjective forenoon, ~4.5 h after lights-on. The male of the *laH *strain, however, showed anticipation of the light-on transition and thereby began activity ~1 h before lights-on. Activity in both males ended after lights-off. The oblique arrow in each actogram on day 11 indicates the initiation of DD. There were three transient cycles before steady-state free running rhythms with τ > 24 h and τ < 24 were established in the *haH *male (A) and the *laH *male (B), respectively. The unimodal activity pattern of the *haH *male was retained in DD, but the bimodal activity pattern of the *laH *male was abolished in DD as the evening peak disappeared immediately after the transfer from LD 12:12 to DD.

**Figure 5:**
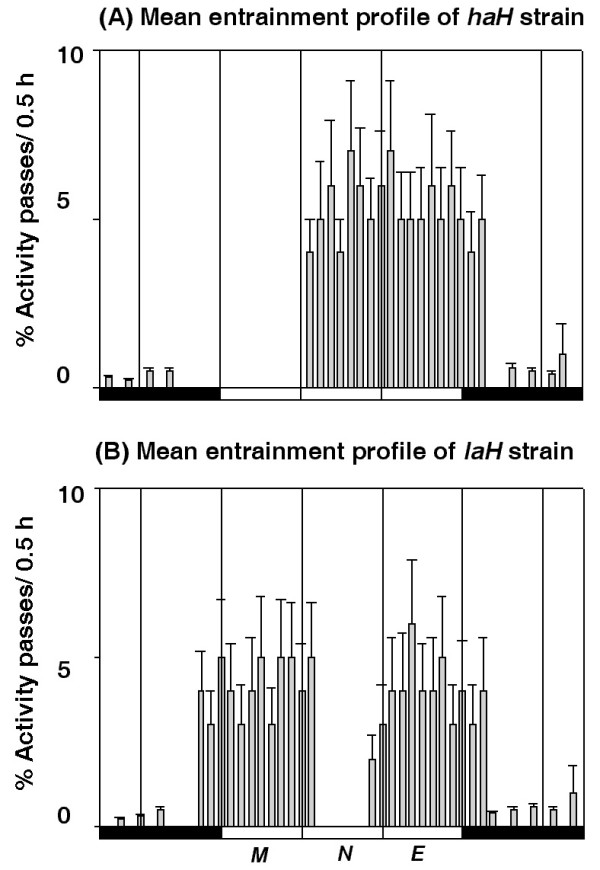
**Mean entrainment activity profile shown in histogram format for 21 males of each strain for 11 days in LD 12:12 cycles at 20 ± 0.5°C under controlled laboratory conditions**. Males of the *haH *strain had a unimodal activity pattern in which activity onset occurred in the subjective forenoon, ~4.5 h after lights-on (A). Males of the *laH *strain had a bimodal activity pattern in which activity onset occurred ~1 h before lights-on (B). The other notations are the same as used in Figure 1.

Both strains of *D. helvetica *were also entrained by LD 12:12 cycles at 20 ± 0.5°C in the laboratory. Figure 4 shows the double plotted activity records of the representative male of each strain and Figure 5 illustrates the mean entrainment pattern of 21 males of each strain for 11 days. Values of Ψ_o_, Ψ_e_, α/ρ ratio and the *TAPC *of both strains are summarized in Table 2. Entrainment parameters of both strains followed the same trend as that exhibited during entrainment to natural LD cycles in the field. Altitude of origin significantly affected Ψ_o_, Ψ_e_, α/ρ and *TAPC *(*p *< 0.01) of these strains in LD 12:12 cycles. Transfers from LD 12:12 cycles to DD were always followed by 2–3 transient cycles before steady state free-running rhythms were established in each strain (Figure 4). Although both strains exhibited free-running rhythmicity with a unimodal activity pattern, the values of τ and *TAPC *differed significantly (*p *< 0.01) (Table 2). The unimodal activity pattern of the *haH *strain was retained during the transient cycles and subsequent steady-state free-runs in DD. However, the bimodal activity pattern was abolished in all males of the *laH *strain immediately after the LD 12:12 to DD transfer, as the evening peak disappeared. The morning peak was retained during transient cycles and in subsequent steady-state free-runs.

**Table 2:** Entrainment and free-running parameters of *D. helvetica *strains in the laboratory

Strain	Ψ_o_	Ψ_e_	α/ρ in LD 12:12	*TAPC *in LD 12:12	α/ρ in DD	τ (h)	*TAPC *in DD
*haH*	4.5 (0.4)	- 1.1 (0.3)	0.7 (0.3)	596 (32)	0.4 (0.2)	26.1 (0.6)	592 (59)
*laH*	-1.3 (0.4)	0.4 (0.2)	1.7 (0.4)	782 (61)	0.4 (0.1)	21.7 (0.4)	377 (32)

Mean values (± *SD*, *N *= 21) for the phase of activity onset (Ψ_o_), phase of activity end (Ψ_e_), the ratio of activity phase to rest phase (α/ρ) and the total activity per cycle (*TAPC*) for males of the *haH *strain and the *laH *strain of *D. helvetica *entrained by LD 12:12 cycles at constant temperature of 20 ± 0.5°C in the laboratory.

## Discussion

Altitude of origin delayed the phase of activity onset of the *haH *strain of *D. helvetica *by ~6 h when compared to that of the *laH *strain in all entraining LD cycles in the field and laboratory. Failure of the *haH *flies to begin activity before sunrise in the field like the *laH *flies (Figure 1) might be due to the masking effect [22] of 'non-permissible' low temperature (~9°C) in the field at sunrise (Figure 2A). This possibility should be ruled out because the 'permissible' temperature at 20°C at sunrise, as imposed in the *CT *regime, failed to advance the Ψ_o _of this strain (Figure 3A). It appears that the lights-on transition of LD cycles was the phase determining photic signal for the *haH *flies to initiate activity as they also commenced activity ~4.5 h after lights-on of LD 12:12 cycles (100 lux during L) (Figure 4A). Even the increment in light intensity of the photophase from 100 to 500 lux or the increment in duration of the photophase from 12 to 14 h failed to advance the Ψ_o _of this strain (in preparation). The *laH *flies, however, initiated activity ~1 h before lights-on of all entraining light-dark cycles. Thus, these results demonstrate that the altitude of origin altered the Ψ_o _of the *haH *strain. Moreover, the lights-on transition appears to be the photic signal of LD cycles for timing the phase of activity onset in both these strains. Three reference points of photophase – namely, the lights-on, lights-off and midpoint – are useful in determining the activity onset in various insects [23].

The activity pattern and the phase of activity onset during entrainment seem to have adaptive relevance in *Drosophila *[8]. The unimodal activity pattern with a delayed phase of activity onset in the *haH *strain of *D. helvetica *may be the result of natural selection to avoid low temperature in the morning at the high altitude of its origin. High altitude Himalayan strains (1,321 to 2,346 m a. s. l.) of *D. ananassae *were also characterized by a unimodal activity pattern with a delayed activity peak occurring late in the morning [8]. Low temperature and decreased barometric pressure at high altitude are reported to reduce walking speed and flight performance in *D. melanogaster *[24]. The *laH *strain of *D. helvetica*, however, showed a bimodal activity pattern in which the early morning peak was separated from the evening peak by a period of ~4 h of inactivity in the noon which should be considered as the result of natural selection so that these flies would take advantage of the relatively cooler part of the day in the morning hours for foraging, mating, etc. but would avoid desiccation on exposure to relatively high temperature at noon at its breeding site in the field. Forty-two low altitudinal strains of *D. ananassae *originating from the western coast of India (0 m, a. s. l.) had bimodal activity patterns with early morning peaks [8]. Females of a *Drosophila *parasitic wasp, *Leptopilina heterostoma*, from southern regions had bimodality with morning and evening activity peaks, while the northern strains were active mostly during the afternoon [25].

Altitude of origin also altered the phase of activity termination of the *haH *strain of *D. helvetica *as it was labile and temperature dependent unlike that of the *laH *strain. The *haH *flies ceased activity about one hour prior to sunset in the *NFT *regime when the environmental temperature was ~14°C (Figure 2A). Early cessation of activity of the *haH *strain in the field might be the result of negative masking by temperature which rapidly falls in the rarefied air at the high altitude of its breeding site. When this strain was subjected to the permissible temperature at 20°C as in the *CT *regime in the field, its activity was extended by ~2 h as compared to that in the *NFT *regime. Similarly, its activity ceased ~1 h after lights-off when entrained by LD 12:12 cycles (100 lux during L) (Figure 4A) or by LD cycles in which the photophase (500 lux during L) varied from 10 to 14 h per 24 h at 20°C (in preparation). These results suggest that the termination of activity of the *haH *strain was labile and temperature dependent. By contrast, the *laH *strain terminated activity ~0.4 h after sunset during entrainment to all natural and artificial LD cycles, as mentioned above, suggesting that its Ψ_e _was rigid.

In order to confirm that the delayed Ψ_o _of the *haH *strain or the advanced Ψ_o _of the *laH *strain of *D. helvetica *were not simply a consequence of an exogenous response to lights-on or lights-off transitions of light-dark cycles, the flies entrained by LD 12:12 cycles were transferred to DD to determine the period of free-running rhythm, which is the most reliable property of the underlying circadian pacemaker. The altitude of origin indeed affected τ of the *haH *strain as it was about 4.5 h longer than that of the *laH *strain (Table 2). In general, an advanced Ψ_o _is correlated with short τ and delayed Ψ_o _with long τ, as predicted from the model formulated for explaining the relationship among eclosion rhythm parameters of *D. pseudoobscura *[26]. The present results on *D*. *helvetica *strains agree with this model, and the difference in Ψ_o _of these strains could be made clear by considering the different τ s of these strains. The *haH *strain with long τ and *laH *strain with short τ are expected to phase lag and phase advance, respectively, when entrained by LD 12:12 cycles. That is exactly what we observed. Altitude of origin also affected the τ of eclosion and oviposition rhythms of *D. ananassae *strains [17,19]. Latitude of origin as well influenced τ of eclosion rhythm of *D. littoralis *[5], *D. subobscura *[7] and *D. ananassae *[9,20,21].

## Conclusion

This appears to be the first report analyzing the effects of high altitude of origin on the circadian locomotor activity of *D. helvetica*. Parameters of entrainment and free-running rhythm of the *haH *strain of *D. helvetica *are unique among known *Drosophila *species. Its delayed but rigid phase of activity onset that refers to the lights-on transition of entraining photophase and the early but labile phase of activity termination that could be postponed by high temperature should be regarded as behavioral adaptations in response to the low temperature and other environmental conditions prevailing at the high altitude of its breeding site which have profoundly influenced the evolution of the pacemaker controlling its locomotor activity rhythm.

## Abbreviations

α, activity phase; α/ρ, ratio of activity phase to rest phase; *CT*, constant temperature; τ, period of free running rhythm under constant conditions; *NFT*, naturally fluctuating temperature; Ψ_o_, phase of activity onset; Ψ_e_, phase of activity end; Ψ_*M-E*_, interval between morning and evening peaks; ρ, rest phase; *TAPC*, total activity passes per cycle

## Competing interests

The author(s) declare that they have no competing interests.

## Authors' contributions

KVL performed the experiments with the help of CV and MKS, and all three prepared the initial draft of the manuscript. SBI fabricated and maintained the *Drosophila *activity units, related hardware and software. MSK and AJS statistically analyzed the data. RJB and DSJ conceived and supervised the experiments. All authors approved the final version of the manuscript.
